# Interventions to optimize duration of antibiotic therapy and encourage oral transition for uncomplicated gram-negative blood stream infections across a health system

**DOI:** 10.1017/ice.2025.10359

**Published:** 2026-02

**Authors:** Jared Olson, Adam Hersh, John Veillette, C. Dustin Waters, Brandon J Webb, Nick Tinker, Stephanie May, Michelle Matheu, Nora Fino, Rachel Foster, Mary Hutton, Erin Stahl, Jena Rhodes, Edward Stenehjem, Andrew Pavia, Payal Patel, Allan Seibert, Whitney Buckel

**Affiliations:** 1 Division of Pediatric Infectious Diseases, Department of Pediatrics, Spencer Fox School of Medicine, University of Utah, Salt Lake City, UT, USA; 2 Department of Pharmacy, Primary Children’s Hospital, Salt Lake City, UT, USA; 3 Infectious Diseases Telehealth Service, Intermountain Health, Murray, UT, USA; 4 Department of Pharmacy, McKay-Dee Hospital, Ogden, UT, USA; 5 Division of Infectious Diseases, Intermountain Medical Center, Murray, UT, USA; 6 Division of Epidemiology, Department of Internal Medicine, University of Utah, Salt Lake City, UT, USA.; 7 Department of Pharmacy, Intermountain Medical Center, Murray, UT, USA; 8 Department of Pharmacy, Utah Valley Hospital, Provo, UT, USA; 9 Pharmacy Services, Intermountain Health, Taylorsville, UT, USA; 10 Department of Medicine, Division of Infectious Diseases, University of Colorado School of Medicine, Aurora, CO, USA; 11 Department of Internal Medicine, Spencer Fox School of Medicine, University of Utah, Salt Lake City, UT, USA

## Abstract

**Background:**

Shorter antibiotic courses and transition to oral therapy for uncomplicated gram-negative bloodstream infections (GN-BSI) are evidence-supported yet remain challenging to implement. Here we report our experience with a GN-BSI antimicrobial stewardship (AS) quality improvement initiative in a large health system.

**Methods:**

We implemented two sequential AS interventions in adult patients hospitalized with uncomplicated GN-BSI: (1) mandatory AS review of patients discharging on intravenous (IV) antibiotics (“OPAT review”) and (2) a clinical guideline informing oral antibiotic transition and duration, in our 22-hospital system. We evaluated the initiative from January 2018 to September 2024. Pre- and postimplementation rates of (1) IV antibiotics at discharge and (2) total length of antibiotic therapy were calculated across the following periods: preintervention, after OPAT review, and after guideline implementation. Secondary outcomes included duration <10 days, oral antibiotic prescribing, and guideline-recommended dosing.

**Results:**

3,231 patients (preintervention: 666, postOPAT review: 1,357, postguideline: 1,208) were included. We observed decreases in IV antibiotics at discharge (22.7% preintervention, 10.7% postOPAT review, and 9.2% postguideline, *p* < 0.001) and median length of treatment (13.5 days preintervention to 10.7 days postguideline, *p* < 0.001). We also observed improvement in durations <10 days (19.1% vs 45%, *p* < 0.001), oral antibiotic prescriptions, and appropriate dosing (2.8% vs 33.5%, *p* < 0.001), but no difference in rates of BSI recurrence, mortality, or *C. difficile* infection.

**Conclusion:**

After implementing GN-BSI-focused AS initiatives in our large health system, we observed a shift toward more frequent oral rather than IV antibiotics at discharge, and shorter overall antibiotic durations, without obvious changes in adverse outcomes.

## Introduction

Historically, standard practice for antibiotic treatment of gram-negative blood stream infections (GN-BSI) included 14 days of intravenous therapy. Subsequently, randomized controlled trials and carefully conducted observational studies have suggested that for uncomplicated GN-BSI, a 7-day course is as effective as a 14-day course when adequate source control has been achieved.^1,2^ Shorter durations of therapy may be associated with decreased rates of multidrug-resistant bacteria, adverse events, superinfections and duration of hospitalization.^2,3^

Similarly, a growing body of evidence now suggests that transition to oral therapy from parenteral antibiotics is associated with similar outcomes compared to full-course intravenous therapy, and has been associated with shorter hospital length of stay.^4^ Although conflicting data exist as to whether oral fluoroquinolones and trimethoprim/sulfamethoxazole (TMP/SMX) have lower recurrence rates compared to oral beta-lactams, given nominal differences in absolute event rates, expert consensus guidelines recommend oral beta-lactams using dosing likely to attain pharmacokinetic-pharmacodynamic targets as appropriate treatment options.^4–12^

To encourage adoption of evidence-based antibiotic prescribing, including shorter antibiotic courses and transition to oral therapy where appropriate, the antibiotic stewardship (AS) collaborative in our large health system implemented a quality improvement initiative focused on improving antibiotic prescribing in adult patients hospitalized with uncomplicated GN-BSI. Here we describe our experience with the implementation of two sequential AS interventions to optimize length of treatment and transition to oral therapy for GN-BSI: (1) mandatory AS review (or infectious disease consultation) for all patients with planned discharge on intravenous (IV) antibiotics (hereafter “OPAT review”—outpatient parenteral antibiotic therapy) and (2) development and dissemination of an institutional guideline providing evidence-based criteria for 7-day duration and oral antibiotic transition for uncomplicated GN-BSI.

## Methods

### Context

Intermountain Health (IH) is a nonprofit health system in the Intermountain West of the United States comprised of 22 adult hospitals in Utah and Idaho at the time of these quality improvement interventions.

Prospective audit and feedback, including real time alerts for positive blood cultures to frontline pharmacists and review by infectious diseases/antimicrobial stewardship (ID/AS) pharmacists Monday through Friday has been in place at IH systemwide since 2015. Creation and dissemination of antimicrobial guidelines for specific conditions, including pneumonia, appendicitis, and urinary tract infections, is another key component of the IH stewardship model.

### OPAT review

The first AS intervention introduced was OPAT review, which established an expectation for ID/AS pharmacists or a consulting infectious disease physician to review all patients prior to discharge on IV antibiotics and involved education, audit and feedback, coordination with care managers and central line teams, and electronic health record (EHR) tools. We conducted a pilot of the OPAT review in one hospital starting July 1, 2018. Rollout of mandatory OPAT review throughout the system started in May 2019 and was fully implemented by October 2019.

### Gram-negative Bacteremia clinical guideline

The second intervention was the development and distribution of an institutional guideline to standardize care and promote evidence-based antibiotic prescribing in adult patients with uncomplicated GN-BSI starting in May 2022. The clinical guideline provided two sets of criteria for oral antibiotic transition and short course antibiotic therapy, respectively. The criteria for oral antibiotic transition required meeting all of the following: (1) achieved clinical stability on IV antibiotics, (2) could reliably take oral antibiotics without impaired absorption, and (3) were not allergic or at high risk for toxicity to an active oral antibiotic. The guideline recommended higher doses of oral beta-lactam antibiotics.^13^ The guideline recommended 7 days of antibiotic therapy for patients meeting all 4 of the following criteria: (1) uncomplicated GN-BSI, defined as known source of bacteremia (including urinary, intra-abdominal/hepatobiliary, skin/soft tissue, pulmonary or central line-associated infections) without a deep-seated complicated infection such as bone/joint, central nervous system, prostate, empyema, deep abscess (eg, renal, liver, epidural), endovascular or hardware infection (eg, mesh, cardiac device, or other synthetic material); (2) Source control achieved; (3) Not severely immunocompromised; and (4) clinical stability achieved by 72 hours on IV antibiotics.^13^ The guideline was published in October 2022 on the internal AS website, distributed to local AS teams and reinforced during local AS program meetings. No specific EHR interventions were used to support the implementation of the guidelines.

### Quality improvement assessment

This institutional evaluation of AS initiatives was reviewed by the Intermountain Health Institutional Review Board and determined to meet criteria for quality improvement prior to data collection and data analysis (IRB #1052004, November 18, 2022). Consistent with this determination, we neither claim nor imply generalizability of results beyond our institution. Sensitivity analyses of data performed using interrupted time series (ITS) were performed post hoc, after initial IRB review, and were conducted with intent to provide more robust insights into the temporal nature of the data and not designed to infer causality.

### Evaluation

We identified patients ≥ 18 years of age with blood cultures collected within the first 3 days of hospitalization (including ED-obtained cultures) between January 2018 and August 2024 with growth of one of the following pathogens: *Escherichia coli*, *Klebsiella* spp, *Enterobacter* spp, *Proteus* spp, *Serratia* spp, *Pseudomonas aeruginosa*, and *Citrobacter* spp during an inpatient hospital stay using the EHR. We excluded patients with hospital length of stay (LOS) ≥ 7 days and those febrile (temperature ≥ 38.3° C) 24 hours prior to hospital discharge. As the intervention was focused on improving care for uncomplicated bacteremia as defined by our institutional guideline, we excluded patients with polymicrobial bacteremia, presence of deep seated infections (defined as presence of International Classification of Diseases Tenth Revision (ICD10) codes [see Supplemental Table 1] for bone/joint/muscle, central nervous system, prostate, complicated pneumonia, endovascular, renal abscess, liver abscess, and device associated infections), multiple discharge antibiotics with the exception of concomitant metronidazole, neutropenia (absolute neutrophil count < 500 cells/mL) within 24 hours of discharge, solid organ or hematopoietic stem cell transplant, total duration of therapy >28 days, discharge to hospice care, transfer to another hospital, or in-hospital death. The analysis was limited to the first episode of GN-BSI per patient.

We categorized patients by the following three periods: preintervention from January 2018 to April 2019; postOPAT review from October 2019 through April 2022; and postguideline from November 2022 through September 2024. We accounted for rolling adoption of the OPAT review and the development and dissemination of the GN-BSI guideline by excluding May to September 2019 and May to October 2022, respectively.

To evaluate hospital variation in planned sub-group analyses, the 22 included hospitals were divided into five groups based on infectious diseases (ID) physician coverage and AS pharmacist assignments. Four large regional medical centers were categorized as “groups” A, B, C, and D, respectively. Each of these centers had over 280 beds (range 284–510), dedicated on-site ID consultation services and one or two full-time ID/AS pharmacists. The fifth group, referred to as “Tele hospitals/Hospital E,” included a smaller urban community hospital with part-time (0.5 FTE) on-site AS pharmacist coverage and 17 additional hospitals with bed sizes between 14 and 150 including 6 critical access hospitals supervised by two full-time remote ID/AS pharmacists and ID consultations remotely by telemedicine ID providers.

We collected the following demographic and clinical variables from the enterprise data warehouse: sex, race, ethnicity, age, hospital site, Charlson comorbidity index, organism causing bacteremia, whether UTI was the source of infection (defined as presence of ICD10 codes N39.0, N10, N12), PITT bacteremia score, intensive care unit (ICU) admission, operative procedure during hospitalization, inpatient antimicrobial use, and outpatient antibiotic prescriptions.

### Outcomes measures

The primary outcomes were the percentage of patients discharged on IV antibiotic therapy and the length of antibiotic therapy, defined as the sum of the discharge prescription length of therapy and the total number of inpatient/ED calendar days during which the patient received a dose of antibiotic. Secondary outcomes included the percentage of patients receiving antibiotic courses less than 8 days, less than 10 days, transition to oral therapy, discharge antibiotic selection, and oral beta lactam dosing.

### Safety outcomes

Safety outcomes included hospital LOS and the following metrics from discharge: 14-day readmission, 14-day ED-revisit, 14-day recurrence of GN-BSI (defined as positive blood culture with the same organism as index infection), 14-day new occurrence of bacteremia, 30-day all-cause mortality, and 30-day *Clostridioides difficile* testing and infection (defined as positive polymerase chain reaction test or toxin assay).

### Primary analysis

Aggregate rates for primary and secondary outcomes were compared across time periods using the Kruskal–Wallis test for continuous data and Fisher’s exact test for categorical data.

### Sensitivity analysis

To better evaluate the temporal relationship of the interventions with changes in the primary outcomes, we conducted a sensitivity analysis using ITS. ITS models were fit using segmented generalized least squares regression with first order autoregressive correlation structure at the system level with a monthly observation period. Models were weighted by number of cases and adjusted for the fraction of cases with UTI, ceftriaxone resistance, ICU admission, *E. coli* and male sex because these variables were likely to influence adherence to the guidelines and varied from month to month. Models estimated two population-level effects: (1) initial change in level, defined as the difference between the observed and counterfactual median or proportion at the start of the intervention; and (2) the change in slope after the intervention. Ljung-Box test was applied to residuals to determine if autocorrelation persisted.

To examine the interventions at the hospital group level, we created a column chart to visualize the percentage of patients receiving discharge IV therapy and boxplots to visualize median total antibiotic duration by intervention period.

Statistical analyses were performed using R statistical software package, version 4.2.3 (R Project for Statistical Computing).

## Results

### Patients

A total of 666 patients were included in the preintervention period, 1357 in the mandatory OPAT review period and 1208 in the sequential post GN-BSI guideline period. Clinical and demographic characteristics are shown in Table 1. Age, sex, and organism distribution were similar between groups. Differences between time periods were observed in ethnicity, UTI as source of infection, Charlson comorbidity index, and PITT bacteremia score. *E coli* was the most common pathogen (>70%) and UTI was a source of infection for >70% of GN-BSI in all groups. Ceftriaxone resistance was observed in less than 10% of cases in all time periods.

**Table 1.** Clinical characteristics by time period

Characteristic	Preintervention *N* = 666	OPAT intervention *N* = 1357	GN bundle intervention *N* = 1208	*P*-value
Hospital groupings, *n* (%)				0.25
Hospital A	75 (11.3)	152 (11.2)	144 (11.9)	
Hospital B	163 (24.5)	275 (20.3)	253 (20.9)	
Hospital C	109 (16.4)	219 (16.1)	173 (14.3)	
Hospital D	89 (13.4)	190 (14.0)	154 (12.7)	
Tele hospitals/hospital E	230 (34.5)	521 (38.4)	484 (40.1)	
Age, median years (IQR)	71 (59, 80)	71 (56, 80)	70 (59, 79)	0.42
Male, *n* (%)	259 (38.9)	514 (37.9)	435 (36.0)	0.42
Race, *n* (%)				0.40
White, *n* (%)	595 (89.3)	1180 (86.9)	1046 (86.6)	
Black, *n* (%)	4 (0.6)	11 (0.8)	14 (1.2)	
Amerindian, *n* (%)	12 (1.8)	24 (1.8)	14 (1.2)	
Pacific islander, *n* (%)	16 (2.4)	46 (3.4)	31 (2.6)	
Multiple	1 (0.2)	1 (0.1)	3 (0.2)	
Asian, *n* (%)	11 (1.7)	20 (1.5)	20 (1.7)	
Unknown/refused, *n* (%)	27 (4.1)	75 (5.5)	80 (6.6)	
Hispanic, *n* (%)	80 (12)	162 (11.9)	185 (15.3)	0.03
Charlson comorbidity index, median (IQR)	6 (3, 10)	6 (3,9)	6 (3,9)	0.02
Organism causing bacteremia, *n* (%)				0.41
*Escherichia coli*, *n* (%)	479 (71.9)	988 (72.8)	880 (72.8)	
*Klebsiella* spp not *aerogenes*, *n* (%)	112 (16.8)	204 (15.0)	171 (14.2)	
*Enterobacter* spp, *n* (%)	12 (1.2)	43 (3.2)	35 (2.9)	
*K. aerogenes*, *n* (%)	10 (1.5)	16 (1.2)	20 (1.7)	
*Pseudomonas aeruginosa*, *n* (%)	21 (3.2)	53 (3.9)	47 (3.9)	
*Citrobacter* spp, *n* (%)	11 (1.7)	19 (1.4)	9 (0.7)	
*Proteus* spp, *n* (%)	18 (2.7)	27 (2)	39 (3.2)	
*Serratia* spp, *n* (%)	3 (0.5)	7 (0.5)	7 (0.6)	
Ceftriaxone resistance, *n* (%)	62 (9.3)	107 (7.9)	100 (8.3)	0.55
Urinary tract infection, *n* (%)	503 (75.5)	956 (70.4)	901 (74.6)	0.02
PITT bacteremia score, median (IQR)	1 (0,3)	1 (0,2)	1 (0,2)	0.02
Intensive care unit admission, *n* (%)	118 (17.7)	267 (19.6)	263 (21.8)	0.10
Operative room visit, *n* (%)	142 (21.3)	294 (21.6)	259 (21.4)	0.98
Total antibiotic duration > 17 days, *n* (%)	38 (5.7)	60 (4.4)	60 (5.0)	0.45

Note. OPAT, outpatient parenteral antibiotic therapy; GN, Gram negative.

**Figure 1.**
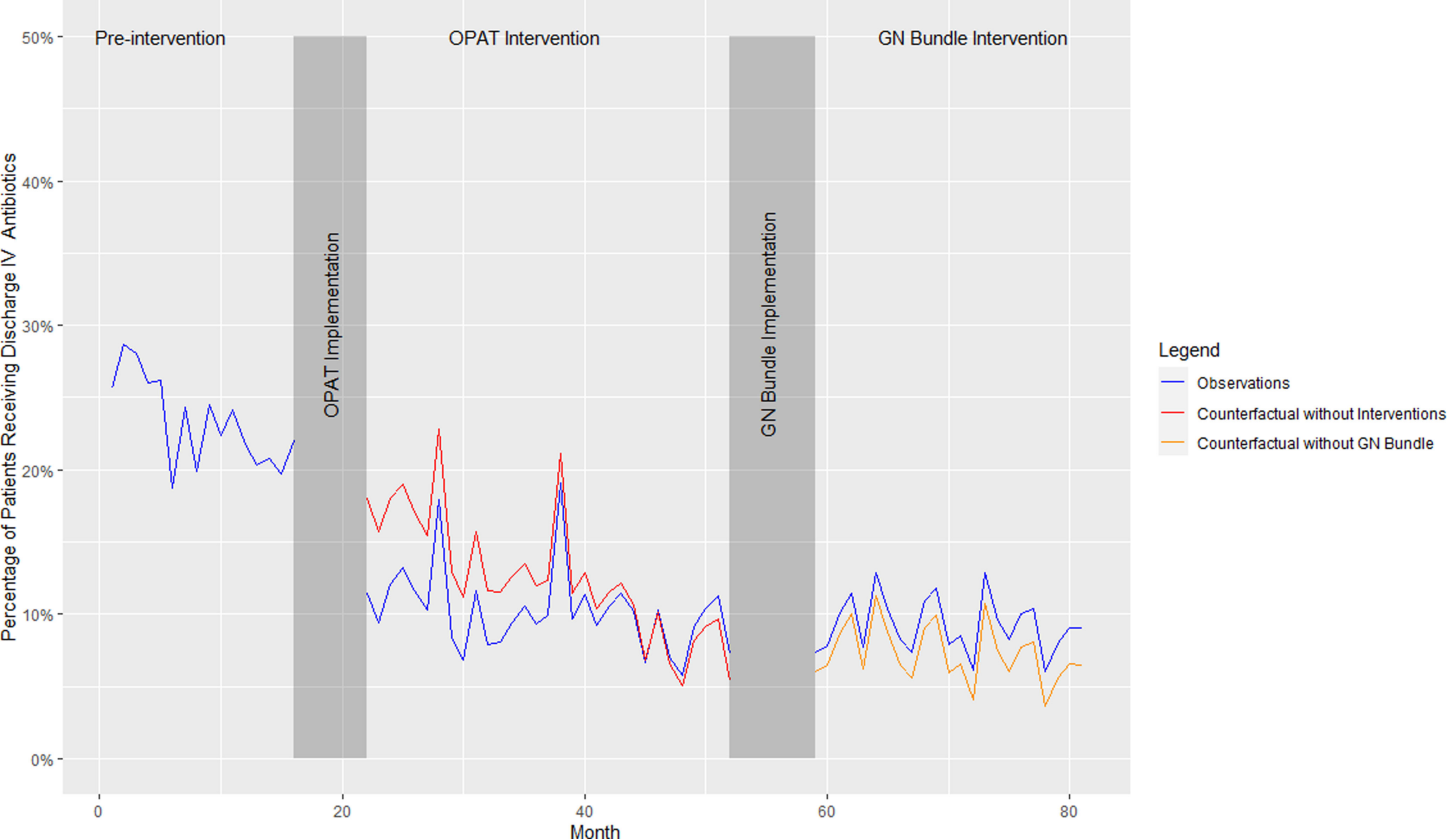
Interrupted time series analysis of discharge IV therapy at system level. *Note:* OPAT, outpatient parenteral antibiotic therapy; GN, Gram negative.

The percentage of patients receiving IV antibiotics at discharge was 22.7% during the preintervention period, 10.7% during the OPAT review period, and 9.2% during the postguideline period (*P* < 0.001) (Table 2). In the ITS sensitivity analysis, implementation of the OPAT review intervention was associated with an initial 6.8% decrease (95% CI [−12.2, −1.4) in discharge IV antibiotics across the system without a significant change in subsequent rate per month (0.27%; 95% CI−0.06, 0.62). No differences were observed in the initial level change (1.25%; 95% CI−0.27%, 5.2%) or subsequent change in rate per month (1.49%; 95% CI−0.19%, 0.31%) associated with dissemination of the GN-BSI guideline (Figure 1). No significant autocorrelation in the model was detected (*p*-value = 0.94 at 24 lags).

**Table 2.** Outcomes by time period

Outcome	Preintervention *N* = 666	OPAT intervention *N* = 1357	GN bundle intervention *N* = 1208	*P*-value
Discharge IV antibiotic, *n* (%)	151 (22.7)	145 (10.7)	111 (9.2)	< 0.001
Oral switch, *n* (%)	515 (77.3)	1196 (88.1)	1080 (89.4)	< 0.001
Total days of therapy, median (IQR)	13.5 (11.0, 15.0)	11.8 (9.0, 14.0)	10.7 (8.6, 13.6)	< 0.001
Received < 10 days of therapy, *n* (%)	127 (19.1)	511 (37.6)	543 (45.0)	< 0.001
Received < 8 days of therapy, *n* (%)	55 (8.3)	239 (17.6)	253 (20.9)	< 0.001
Days of IV therapy, median (IQR)	2.8 (2.0, 5.6)	2.8 (2,4.0)	2.8 (2, 4)	0.37
Follow up blood cultures, *n* (%)	15 (2.3)	21 (1.5)	14 (1.2)	0.19
Length of hospitalization, median (IQR)	4 (3,4)	3 (3,4)	3 (3,4)	0.79
14-day readmission, *n* (%)	25 (3.8)	55 (4.1)	52 (4.3)	0.84
14-day emergency department revisit, *n* (%)	27 (4.1)	71 (5.2)	57 (4.7)	0.50
14 -day recurrence of bacteremia, *n* (%)	3 (0.5)	5 (0.4)	3 (0.2)	0.75
14-day new occurrence bacteremia, *n* (%)	0 (0)	4 (0.3)	4 (0.3)	0.35
30-day mortality, *n* (%)	5 (0.8)	24 (1.8)	19 (1.6)	0.20
30-day *C. difficile* testing, *n* (%)	19 (2.9)	33 (2.4)	28 (2.3)	0.77
30-day *C. difficile* positive, *n* (%)	8 (1.2)	16 (1.2)	8 (0.7)	0.35

Note. OPAT, outpatient parenteral antibiotic therapy; GN, Gram negative.

**Figure 2.**
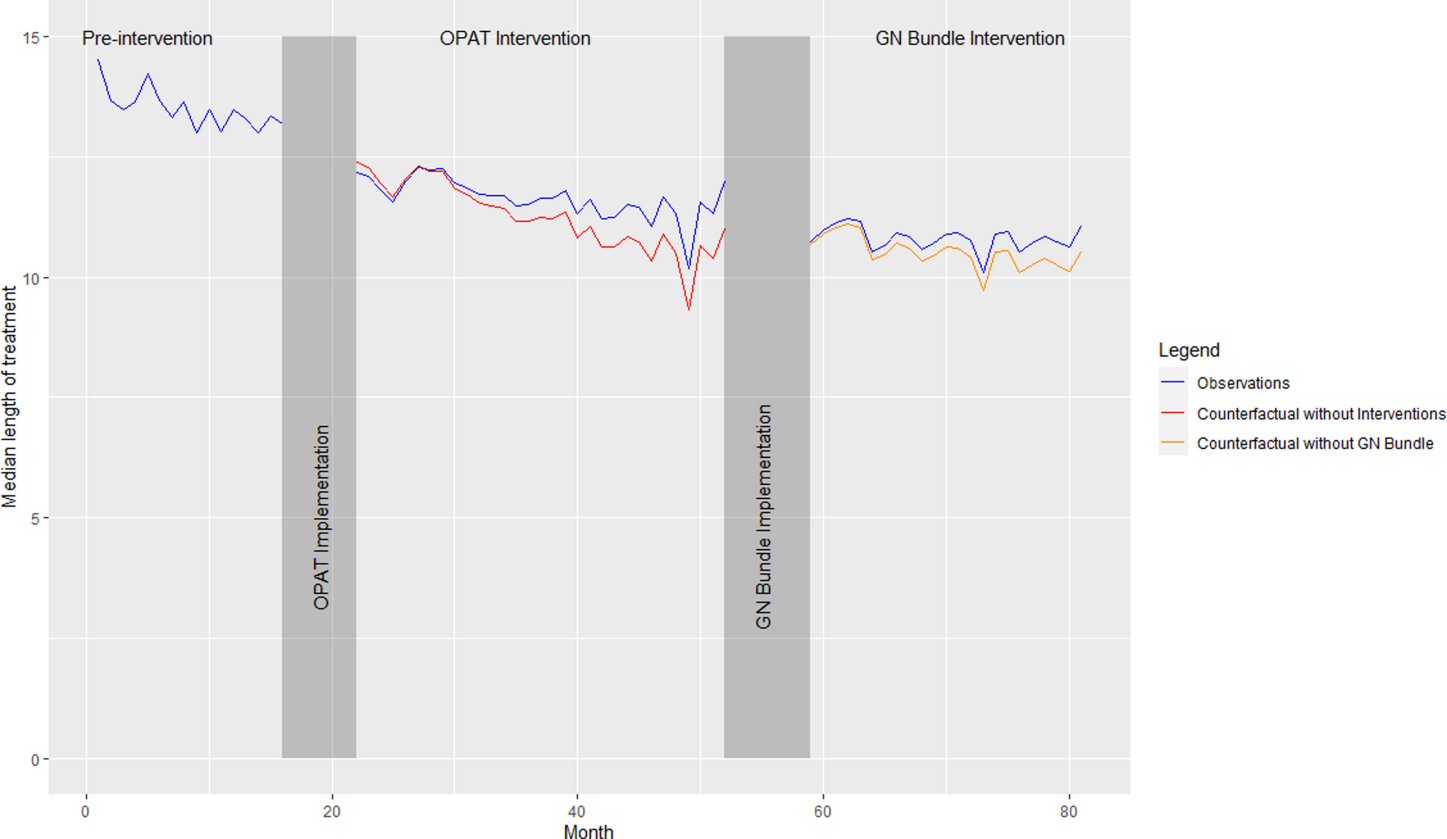
Interrupted time series analysis of median length of treatment at system level. *Note:* OPAT, outpatient parenteral antibiotic therapy; GN, Gram negative.

In aggregate, the median length of therapy was 13.5 days (IQR 11, 15) during the preimplementation period, 11.8 days (IQR 9, 14) during the OPAT review period, and 10.7 days (8.6, 13.6) during the GN-BSI guideline period (*P* < 0.001). The sequential introductions of the mandatory OPAT review and GN-BSI guidelines were not associated with significant estimated changes in level (−0.25 days [95% CI−1.49, 0.98]; 0.03 days [95% CI−0.92, 0.99], respectively) or estimated changes in slope in the ITS sensitivity analysis (0.04 days per month [95% CI−0.04, 0.12]; 0.02 [95% CI−0.04, 0.08], respectively) (Figure 2). No significant autocorrelation in the model was detected (*p*-value = 0.62 at 24 lags).

The percentage of patients receiving < 8 days of therapy was 8.3%, 17.6%, 20.9% (*p* < 0.001) in the preintervention, mandatory OPAT review and sequential GN-BSI periods respectively, while the percentage of patients receiving < 10 days of antibiotic therapy in these periods was 19.1%, 37.6%, and 45.0% (*p* < 0.001).

Changes in discharge oral antibiotic selection were observed between periods (Table 3). Notable observations included increases in tmp/smx (5.1%, 12.5%, and 12.5% in each period, respectively; *P* < 0.001) and cephalexin (3.3%, 12.5%, 16.1%; *p* < 0.001) prescriptions. We also observed reductions in fluoroquinolones (49.1%, 45.4%, 42.5%; *p* 0.02) and cefuroxime prescriptions (8.3%, 2.4%, 1.5%; *p* < 0.001). Recommended dosing adherence for discharge oral beta-lactam prescriptions was 2.8% in the preimplementation period, 19.5% in the OPAT review period, and 33.5% in the postguideline period (*p* < 0.001).

**Table 3.** Discharge antibiotics

Antibiotic selection	Preintervention *N* = 666	OPAT intervention *N* = 1357	GN bundle intervention *N* = 1208	*P*-value
IV Beta-lactam, *n* (%)	158 (23.7)	154 (11.3)	120 (9.9)	< 0.001
Carbapenem or ceftazidime/avibactam, *n* (%)	50 (7.5)	70 (5.2)	70 (5.8)	0.11
Oral fluoroquinolone, *n* (%)	327 (49.1)	617 (45.4)	513 (42.5)	0.02
Oral TMP/SMX, *n* (%)	34 (5.1)	170 (12.5)	151 (12.5)	< 0.001
Amoxicillin, *n* (%)	13 (2)	45 (3.3)	62 (5.1)	0.001
Amoxicillin/clavulanate, *n* (%)	38 (5.2)	110 (8.1)	107 (8.9)	0.05
Cefdinir, *n* (%)	17 (2.6)	36 (2.7)	19 (1.6)	0.15
Cephalexin, *n* (%)	22 (3.3)	170 (12.5)	194 (16.1)	< 0.001
Cefuroxime, *n* (%)	55 (8.3)	33 (2.4)	18 (1.5)	< 0.001
No discharge antibiotic, *n* (%)	0 (0)	19 (1.4)	20 (1.7)	0.005
Consensus recommended beta lactam dosing, n/n beta lactam prescription (%)^a^	4/145 (2.8)	77/394 (19.5)	134/400 (33.5)	< 0.001

Note.

a Dosing assessed regardless of renal function.

OPAT, outpatient parenteral antibiotic therapy; GN, Gram negative.

No differences between groups were observed in hospital LOS, 14-day readmissions, 14-day ED revisits, 14-day recurrence of bacteremia, 14-day new occurrence of bacteremia, 30-day all-cause mortality, 30-day *C difficile* testing, or 30-day *C difficile* infection (Table 2).

### Subgroup analyses

**Figure 3.**
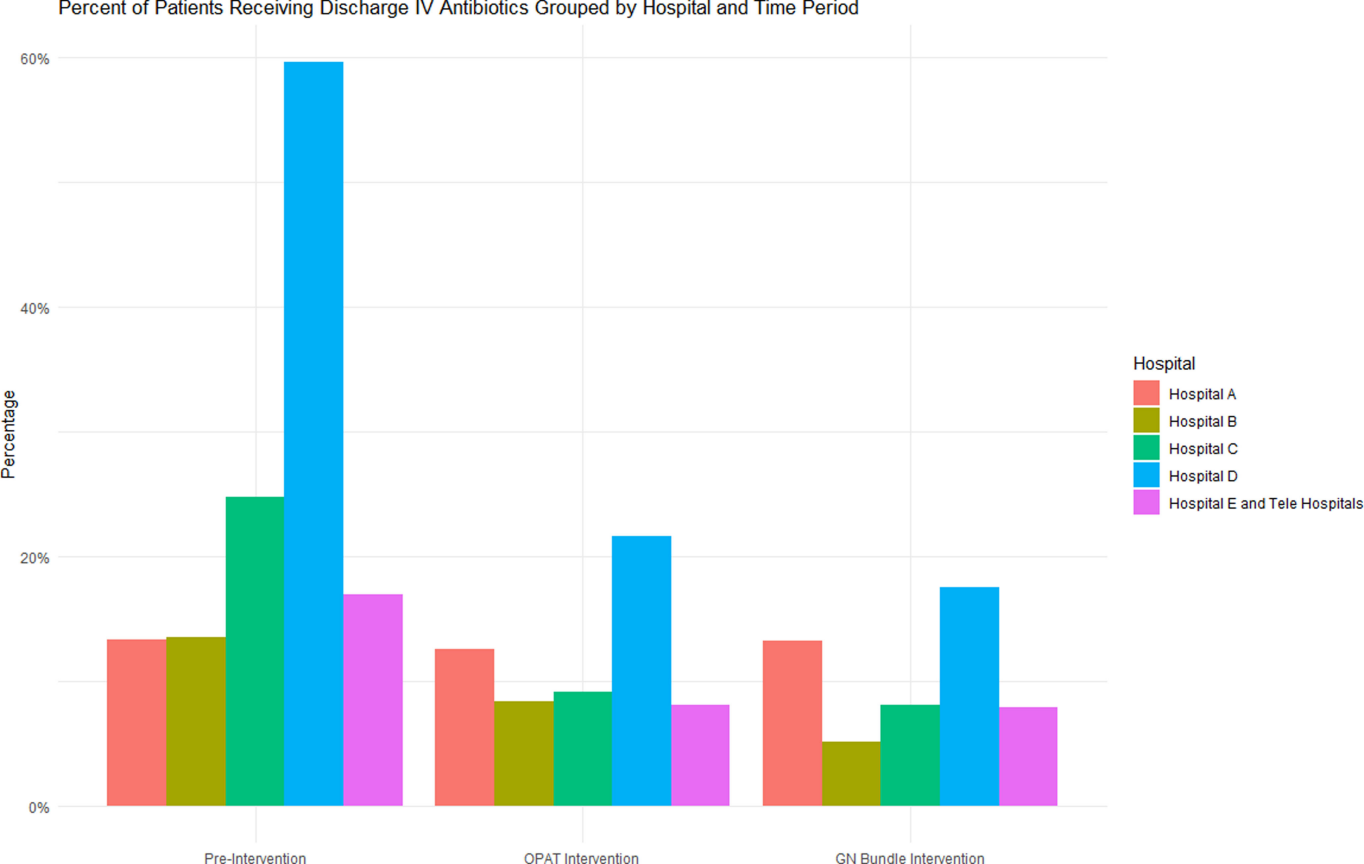
Column chart of discharge IV therapy at hospital group level. *Note:* OPAT, outpatient parenteral antibiotic therapy; GN, Gram negative; IV, intravenous. Bars represent the percentage of patients at each hospital that received discharge IV antibiotics.

**Figure 4.**
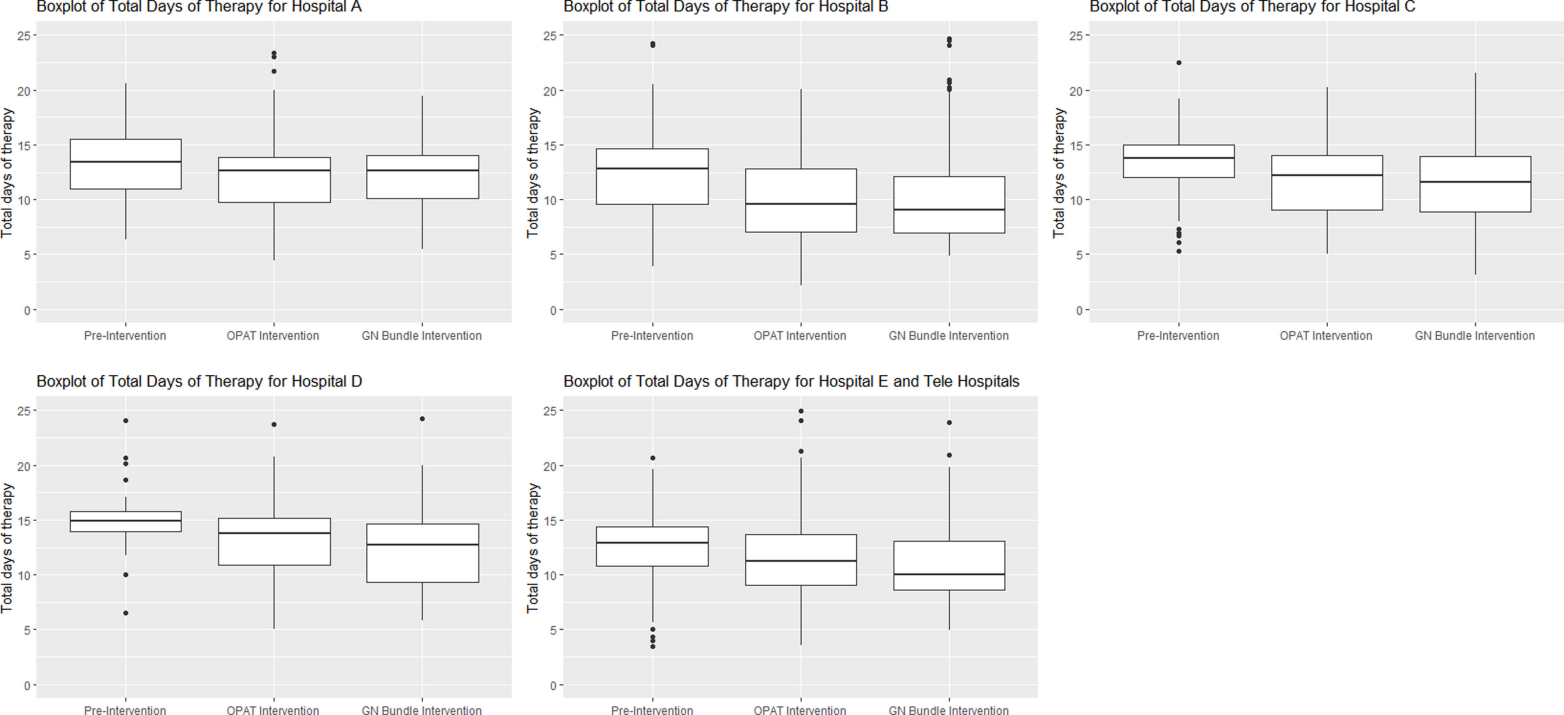
Boxplot of median length of therapy at hospital group level. *Note:* OPAT, outpatient parenteral antibiotic therapy; GN, Gram negative.

There was substantial variation between hospital groups in their use of OPAT and length of therapy at baseline and in response to interventions over time as shown in Figures 3 and 4. These differences appear substantially smaller after the interventions.

### Discussion

When new data emerge to change optimal therapy, it may take many years to change established behaviors.^14^ Here we report the evaluation of two sequential AS interventions for GN-BSI focused on reducing discharge IV treatment and total antibiotic duration in a large healthcare system. We observed a significant decrease in the percentage of patients receiving IV antibiotic therapy at discharge (from 22.7% to 9.2%). The ITS sensitivity analysis suggests that the OPAT review resulted in an immediate decrease in discharge IV antibiotic use. We also observed a significant decrease in the overall median length of antibiotic therapy (from 13.5 to 10.7 days), however the results of our ITS analysis suggest these observations were due to secular trends. Importantly, we did not observe any difference in safety outcomes before or after each intervention implementation. We observed substantial variation between hospitals in baseline rates of IV discharge antibiotics and duration of therapy, as well as in observed response to AS interventions.

Previous studies have suggested that OPAT review by AS/ID providers may have value in decreasing the number of inappropriate courses of OPAT, improving safety, and decreasing costs by identifying patients for whom oral antibiotics are an appropriate alternative.^15,16^ Indeed, this was the experience in our system.

Although we observed reductions in IV antibiotic prescribing at discharge and overall length of therapy over the course of our AS initiatives, we are unable to confirm a temporal association between individual interventions and outcomes, outside OPAT review. It is possible that the more passive nature of the interventions targeting duration or the robust AS review of these patients throughout the study reduced the effect sizes of the tested interventions.

Additional opportunities within our system to further reduce total antibiotic duration exist. At the end of our postintervention period, median LOT was still 10 days. Possible reasons for prolonged durations of therapy include prescribers not counting in-hospital antibiotic duration in total antibiotic duration, discharge outpatient prescriptions with set interval durations of therapy for a full course of antibiotics requiring editing to shorten durations, older references recommending 10 days for pyelonephritis when using beta-lactams or TMP/SMX, inadvertent inclusion of complicated cases in our cohort, and lack of awareness of or disagreement with our guideline.^17^ Our institutional UTI guideline active during these QI initiatives recommended 10 days of therapy for pyelonephritis when using TMP/SMX and cephalosporins, and may have contributed to this observation. This same concern has been raised by other institutions.^18^ We hope the recently published national guideline for complicated UTI will improve adherence to 7-day durations given pyelonephritis is the most common source contributing to GN-BSI.^19^

In AS, proven interventions often have differing results when applied to different settings. Within our large health system, we observed substantial variation across sites in baseline practices and in the observed outcomes after interventions. Deeper understanding of the reasons for variation in the willingness to implement new evidence-based practices and the behavioral strategies that are most effective remain key areas for future research. It is our experience that having a functional AS quality improvement (QI) team alone may be insufficient to drive change. Change readiness and change capacity are important factors that determine QI initiative success.^20–23^ Despite implementing coordinated AS interventions at the system level, we still observed variation in antibiotic prescribing patterns at the local hospital levels. Whether these differences were due to local culture and readiness, differences in engagement of key thought leaders, negotiation skills of the AS teams, or other factors, our observations highlight the importance of considering potential unique local dynamics when implementing QI interventions across a large health system.

Our findings should be considered in light of several limitations. First, findings from our evaluation of an institutional quality improvement initiative cannot be generalized to other settings where AS program structure and resources or clinical practice patterns may be different. Second, we relied on readily available electronic data including ICD10 diagnosis codes to approximate patients with uncomplicated GN-BSI. Due to potential misclassifications, patients with complicated GN-BSI may have been included, which may have had some influence on the observed outcomes in what we intended to be our target population. Third, observations may have likewise been influenced by the use of total duration of antibiotic therapy instead of duration of active antimicrobial therapy. However, given that ceftriaxone is our preferred empiric antimicrobial and ceftriaxone resistance was less than 10% in our cohort, this is unlikely to have changed median length of treatment. Fourth, due to the retrospective design, it is possible that some clinical outcomes may have been misclassified (eg, mortality, readmissions) although we would expect this to be similarly distributed over time. Fifth, extending our preintervention period may have more accurately estimated preintervention trends. Unfortunately, 2018 was the first-year post implementation of a new EHR throughout our health system. We selected 2018 as the beginning of our preintervention period to reduce the possibility of differences between EHRs; most importantly the increased ability to capture outpatient prescriptions in the new EHR.

After implementing two GN-BSI-focused AS initiatives in our large health system, we observed a shift in antibiotic prescribing patterns, to more frequent oral rather than IV antibiotics at discharge, and shorter overall antibiotic durations, without obvious changes in adverse outcomes. Observed prescribing patterns varied by hospital, highlighting the need to identify behavioral and cultural barriers to change.

## Supporting information

Olson et al. supplementary material Olson et al. supplementary material

## Supplementary material

To view supplementary material for this article, please visit https://doi.org/10.1017/ice.2025.10359.

## References

[1] Yahav D, Franceschini E, Koppel F, et al. Seven versus 14 days of antibiotic therapy for uncomplicated gram-negative bacteremia: a noninferiority randomized controlled trial. Clin Infect Dis 2019;69:1091–1098. 30535100 10.1093/cid/ciy1054

[2] Chotiprasitsakul D, Han JH, Cosgrove SE, et al. Comparing the outcomes of adults with Enterobacteriaceae bacteremia receiving short-course versus prolonged-course antibiotic therapy in a multicenter, propensity score-matched cohort. Clin Infect Dis 2018;66:172–177. 29190320 10.1093/cid/cix767 PMC5849997

[3] Corey GR, Stryjewski ME, Everts RJ. Short-course therapy for bloodstream infections in immunocompetent adults. Int J Antimicrob Agents 2009;34:S47–S51. 19931818 10.1016/S0924-8579(09)70567-9

[4] Mercuro NJ, Stogsdill P, Wungwattana M. Retrospective analysis comparing oral stepdown therapy for enterobacteriaceae bloodstream infections: fluoroquinolones versus β-lactams. Int J Antimicrob Agents 2018;51:687–692. 29284155 10.1016/j.ijantimicag.2017.12.007

[5] Alzaidi S, Veillette JJ, May SS, et al. Oral β-lactams, fluoroquinolones, or trimethoprim-sulfamethoxazole for definitive treatment of uncomplicated Escherichia coli or Klebsiella species bacteremia from a urinary tract source. Open Forum Infect Dis 2024;11:ofad657. 38370295 10.1093/ofid/ofad657 PMC10873539

[6] Veillette JJ, May SS, Alzaidi S, et al. Real-world effectiveness of intravenous and oral antibiotic stepdown strategies for gram-negative complicated urinary tract infection with bacteremia. Open Forum Infect Dis 2024;11:ofae193. 38665174 10.1093/ofid/ofae193 PMC11045028

[7] Dore M, Duffy R, Caputo L, et al. Transition to oral beta-lactam therapy in uncomplicated gram-negative bacteremia: a systematic review and meta-analysis. J Hosp Med 2025;20:866–873. doi: 10.1002/jhm.70041. 40183641

[8] Sutton JD, Stevens VW, Chang NCN, Khader K, Timbrook TT, Spivak ES. Oral β-lactam antibiotics vs fluoroquinolones or trimethoprim-sulfamethoxazole for definitive treatment of enterobacterales bacteremia from a urine source. JAMA Netw Open 2020;3:e2020166. 33030555 10.1001/jamanetworkopen.2020.20166 PMC7545306

[9] Punjabi C, Tien V, Meng L, Deresinski S, Holubar M. Oral fluoroquinolone or trimethoprim-sulfamethoxazole vs. beta-lactams as step-down therapy for Enterobacteriaceae bacteremia: systematic review and meta-analysis. Open Forum Infect Dis 2019;6:ofz364. 31412127 10.1093/ofid/ofz364 PMC6785705

[10] McAlister MJ, Rose DT, Hudson FP, Padilla-Tolentino E, Jaso TC. Oral β-lactams vs fluoroquinolones and trimethoprim/sulfamethoxazole for step-down therapy for Escherichia coli, Proteus mirabilis, and Klebsiella pneumoniae bacteremia. Am J Health Syst Pharm 2023;80:S33–S41. 35868628 10.1093/ajhp/zxac202

[11] Kutob LF, Justo JA, Bookstaver PB, Kohn J, Albrecht H, Al-Hasan MN. Effectiveness of oral antibiotics for definitive therapy of Gram-negative bloodstream infections. Int J Antimicrob Agents 2016;48:498–503. 27590704 10.1016/j.ijantimicag.2016.07.013

[12] Arensman K, Shields M, Beganovic M, et al. Fluoroquinolone versus beta-lactam oral step-down therapy for uncomplicated streptococcal bloodstream infections. Antimicrob Agents Chemother 2020;64:e01515–e01520. 32839223 10.1128/AAC.01515-20 PMC7577143

[13] Heil EL, Bork JT, Abbo LM, et al. Optimizing the management of uncomplicated gram-negative bloodstream infections: consensus guidance using a modified Delphi process. Open Forum Infect Dis 2021;8:ofab434. 34738022 10.1093/ofid/ofab434 PMC8561258

[14] Morris ZS, Wooding S, Grant J. The answer is 17 years, what is the question: understanding time lags in translational research. J R Soc Med 2011;104:510–520. doi: 10.1258/jrsm.2011.110180. 22179294 PMC3241518

[15] Sharma R, Loomis W, Brown RB. Impact of mandatory inpatient infectious disease consultation on outpatient parenteral antibiotic therapy. Am J Med Sci 2005;330:60–64. 16103785 10.1097/00000441-200508000-00002

[16] Heintz BH, Halilovic J, Christensen CL. Impact of a multidisciplinary team review of potential outpatient parenteral antimicrobial therapy prior to discharge from an academic medical center. Ann Pharmacother 2011;45:1329–1337. 21990938 10.1345/aph.1Q240

[17] Gupta K, Hooton TM, Naber KG, et al. Infectious diseases society of America; European society for microbiology and infectious diseases. international clinical practice guidelines for the treatment of acute uncomplicated cystitis and pyelonephritis in women: a 2010 update by the Infectious Diseases Society of America and the European Society for Microbiology and Infectious Diseases. Clin Infect Dis 2011;52:e103–e120. 21292654 10.1093/cid/ciq257

[18] DiPietro J, Dubrovskaya Y, Marsh K, et al. Antibiotic stewardship bundle for uncomplicated gram-negative bacteremia at an academic health system: a quasi-experimental study. Antimicrob Steward Healthc Epidemiol 2024;4:e171. 39411661 10.1017/ash.2024.395 PMC11474889

[19] Trautner BW et al. Complicated urinary tract infections (cUTI): clinical guidelines for treatment and management. IDSA. 2025. (https://www.idsociety.org/practice-guideline/complicated-urinary-tract-infections).

[20] Madu A. Challenges in conducting quality improvement projects: reflections of a junior doctor. Future Healthc J 2022;9:333–334. 36561816 10.7861/fhj.2022-0076 PMC9761457

[21] Livorsi DJ, Drainoni ML, Reisinger HS, et al. Leveraging implementation science to advance antibiotic stewardship practice and research. Infect Control Hosp Epidemiol 2022;43:139–146. 34852212 10.1017/ice.2021.480

[22] Elango S, Szymczak JE, Bennett IM, Beidas RS, Werner RM. Changing antibiotic prescribing in a primary care network: the role of readiness to change and group dynamics in success. Am J Med Qual 2018;332:154–161. 10.1177/1062860617716541 PMC6348112 28728423

[23] Thampi N, Szymczak JE, Leis JA. Applying behavioral frameworks to antimicrobial stewardship. Infect Control Hosp Epidemiol 2020;41:628–630. doi: 10.1017/ice.2020.40. 32279666

