# Supplementary material for: Interventions to optimize duration of antibiotic therapy and encourage oral transition for uncomplicated gram-negative blood stream infections across a health system
Source: Infect Control Hosp Epidemiol. 2025 Nov 20;47(2):180–8. doi: 10.1017/ice.2025.10359 (PMC12926339; doi:10.1017/ice.2025.10359)
Supplement: Olson et al. supplementary material [file S0899823X25103590sup001.docx]

Supplement table 1: ICD-10 Codes used to create diagnostic groups

| Diagnostic group | ICD-10 Codes |
| --- | --- |
| Musculoskeletal infections | M00 (Pyogenic Arthritis),  M46.2 (Osteomyelitis of vertebra),  M46.3 (Infection of intervertebral disk),  M46.4 (Discitis, unspecified),  M60.0 (Infective Myositis),  M65.0 (Abscess of tendon sheath),  M65.1 (Other infective [teno]synovitis),  M71.0 (Abscess of bursa),  M71.1 (Other infective bursitis),  M72.6 (necrotizing fasciitis),  M86.0 (Acute hematogenous osteomyelitis),  M86.1 (Other acute osteomyelitis),  M86.2 (Subacute osteomyelitis,  M86.4 (Chronic osteomyelitis with draining sinus),  M86.5 (Other chronic hematogenous osteomyelitis),  M86.6 (Other chronic osteomyelitis),  M86.8 (Other osteomyelitis),  M86.9 (Osteomyelitis, unspecified) |
| Central nervous system infections | G00 (Bacterial meningitis, not elsewhere classified),  G01 (Meningitis in bacterial diseases classified elsewhere),  G04.2 (Bacterial meningoencephalitis and meningomyelitis, not elsewhere classified),  G06 (Intracranial and intraspinal abscess and granuloma),  G07 (Intracranial and intraspinal abscess and granuloma in diseases classified elsewhere) |
| Prostatitis | N41.0 (Acute prostatitis),  N41.1 (Chronic prostatitis),  N41.2 (Abscess of prostate),  N41.3 (Prostatocystitis) |
| Pneumonia, complicated | J85.0 (Gangrene and necrosis of lung),  J85.1 (Abscess of lung with pneumonia),  J85.2 (Abscess of lung without pneumonia),  J85.3 (Abscess of mediastinum),  J86.0 (Pyothorax with fistula),  J86.9 (Pyothorax without fistula) |
| Endovascular infections | I30.1 (Infective pericarditis),  I33 (Acute and subacute endocarditis),  I38 (Endocarditis, valve unspecified),  I39 (Endocarditis and heart valve disorders in diseases classified elsewhere) |
| Liver abscess | K75.0 (Abscess of liver) |
| Renal abscess | N15.1 (renal and perinephric abscess) |
| Device associated | T82.6 (Infection and inflammatory reaction due to cardiac valve prosthesis),  T82.7 (Infection and inflammatory reaction due to other cardiac and vascular devices, implants and grafts),  T83.5 (Infection and inflammatory reaction due to prosthetic device, implant and graft in urinary system),  T8.6 (Infection and inflammatory reaction due to prosthetic device, implant and graft in genital tract),  T84.5 (Infection and inflammatory reaction due to internal joint prosthesis),  T84.6 (Infection and inflammatory reaction due to internal fixation device),  T84.7 (Infection and inflammatory reaction due to other internal orthopedic prosthetic devices, implants and grafts),  T85.7 (Infection and inflammatory reaction due to other internal prosthetic devices, implants and grafts) |
| Urinary tract infection | N10 (Acute pyelonephritis),  N12 (Tubulo-interstitial nephritis, not specified as acute or chronic),  N30.00 (Acute cystitis without hematuria),  N30.01 (Acute cystitis with hematuria),  N30.80 (Other cystitis without hematuria),  N30.81 (Other cystitis with hematuria),  N30.9 (Cystitis, unspecified),  N39.0 (Urinary tract infection, site not specified) |
| Stem cell transplant | T86.00 (Unspecified complication of bone marrow transplant),  T86.01 (Bone marrow transplant rejection),  T86.02 (Bone marrow transplant failure),  T86.03 (Bone marrow transplant infection),  T86.09 (Other complications of bone marrow transplant),  Z94.81 (Bone marrow transplant status),  Z94.84 (Stem cells transplant status) |
| Solid organ transplant | \| T86.10 (Unspecified complication of kidney transplant), \| \| --- \| \| T86.11 (Kidney transplant rejection), \| \| T86.12 (Kidney transplant failure), \| \| T86.13 (Kidney transplant infection), \| \| T86.19 (Other complication of kidney transplant), \| \| T86.20 (Unspecified complication of heart transplant), \| \| T86.21 (Heart transplant rejection), \| \| T86.22 (Heart transplant failure), \| \| T86.23 (Heart transplant infection), \| \| T86.290 (Cardiac allograft vasculopathy), \| \| T86.298 (Other complications of heart transplant), \| \| T86.30 (Unspecified complication of heart-lung transplant), \| \| T86.31 (Heart-lung transplant rejection), \| \| T86.32 (Heart-lung transplant failure), \| \| T86.33 (Heart-lung transplant infection), \| \| T86.39 (Other complications of heart-lung transplant), \| \| T86.40 (Unspecified complication of liver transplant), \| \| T86.41 (Liver transplant rejection), \| \| T86.42 (Liver transplant failure), \| \| T86.43 (Liver transplant infection), \| \| T86.49 (Other complication of liver transplant), \| \| T86.5 (Complications of stem cell transplant), \| \| T86.810 (Lung transplant rejection), \| \| T86.811 (Lung transplant failure), \| \| T86.812 (Lung transplant infection), \| \| T86.818 (Other complications of lung transplant), \| \| T86.819 (Unspecified complication of lung transplant), \| \| T86.850 (Intestine transplant rejection), \| \| T86.851 (Intestine transplant failure), \| \| T86.852 (Intestine transplant infection), \| \| \| T86.858 (Other complication of intestine transplant),  Z94.82 (Intestine transplant status), \| \| --- \| \| Z94.83 (Pancreas transplant status), \| \| Z94.84 (Stem cells transplant status), \| \| \| T86.859 (Unspecified complication of intestine transplant), \| \| T86.890 (Other transplanted tissue rejection), \| \| T86891 (Other transplanted tissue failure), \| \| T86892 (Other transplanted tissue infection), \| \| T86898 (Other complications of other transplanted tissue), \| \| T86899 (Unspecified complication of other transplanted tissue), \| \| T8690 (Unspecified complication of unspecified transplanted organ and tissue), \| \| T8691 (Unspecified transplanted organ and tissue rejection), \| \| T8692 (Unspecified transplanted organ and tissue failure), \| \| T8699 (Other complications of unspecified transplanted organ and tissue), \| \| Z940 (Kidney transplant status), \| \| Z941 (Heart transplant status), \| \| Z942 (Lung transplant status), \| \| Z943 (Heart and lungs transplant status), \|   Z944 (Liver transplant status) |
